# Single- or double-unit UCBT following RIC in adults with AL: a report from Eurocord, the ALWP and the CTIWP of the EBMT

**DOI:** 10.1186/s13045-017-0497-9

**Published:** 2017-06-21

**Authors:** Frédéric Baron, Annalisa Ruggeri, Eric Beohou, Myriam Labopin, Mohamad Mohty, Didier Blaise, Jan J Cornelissen, Patrice Chevallier, Guillermo Sanz, Eefke Petersen, Bipin N Savani, Eliane Gluckman, Arnon Nagler

**Affiliations:** 1 Department of Hematology, University of Liege, CHU Sart-Tilman, 4000 Liege, Belgium; 2 Eurocord, Hospital Saint Louis, AP-HP, and IUH University Paris VII, Paris, France; 3 AP-HP, Hématologie Clinique et Thérapie Cellulaire, Hôpital Saint-Antoine, Paris, France; 4 EBMT Paris Office, Hospital Saint Antoine, Paris, France; 5 Department of Hematology, Institut Paoli Calmettes, Marseille, France; 6 Erasmus Medical Center-Daniel den Hoed Cancer Center, Rotterdam, The Netherlands; 7 Department of Hematology, CHU Nantes, Nantes, France; 8 Servicio de Hematologia, Hospital Universitario La Fe, Valencia, Spain; 9 Department of Hematology, University Medical Centre, Utrecht, The Netherlands; 10 Vanderbilt University Medical Center, Nashville, TN USA; 11 Eurocord, Hospital Saint Louis, AP-HP, and IUH University Paris VII, France Monacord, Centre Scientifique de Monaco, Monaco, Monaco; 12 Division of Hematology and Bone Marrow Transplantation, The Chaim Sheba Medical Center, Tel-Hashomer, Ramat-Gan, Israel and the EBMT Paris Office, Hospital Saint Antoine, Paris, France

**Keywords:** Unrelated cord blood, UCB, Single, Double, AML, ALL, Reduced-intensity, Transplantation

## Abstract

**Background:**

The feasibility of cord blood transplantation (CBT) in adults is limited by the relatively low number of hematopoietic stem/progenitor cells contained in one single CB unit. The infusion of two CB units from different partially HLA-matched donors (double CBT) is frequently performed in patients who lack a sufficiently rich single CB unit.

**Methods:**

We compared CBT outcomes in patients given single or double CBT following reduced-intensity conditioning (RIC) in a retrospective multicenter registry-based study. Inclusion criteria included adult (≥18 years) patients, acute myeloid leukemia (AML) or acute lymphoblastic leukemia (ALL), complete remission (CR) at the time of transplantation, first single (with a cryopreserved TNC ≥ 2.5 × 10^7^/kg) or double CBT between 2004 and 2014, and RIC conditioning.

**Results:**

Data from 534 patients with AML (*n* = 408) or ALL (*n* = 126) receiving a first single (*n* = 172) or double (*n* = 362) CBT were included in the analyses. In univariate analysis, in comparison to patients transplanted with a single CB, double CB recipients had a similar incidence of neutrophil engraftment but a suggestion for a higher incidence of grade II–IV acute GVHD (36 versus 28%, *P* = 0.08). In multivariate analyses, in comparison to single CBT recipients, double CBT patients had a comparable incidence of relapse (HR = 0.9, *P* = 0.5) and of nonrelapse mortality (HR = 0.8, *P* = 0.3), as well as comparable overall (HR = 0.8, *P* = 0.17), leukemia-free (HR = 0.8, *P* = 0.2) and GVHD-free, relapse-free (HR = 1.0, *P* = 0.3) survival.

**Conclusions:**

These data failed to demonstrate better transplantation outcomes in adult patients receiving double CBT in comparison to those receiving single CBT with adequate TNC after RIC.

**Electronic supplementary material:**

The online version of this article (doi:10.1186/s13045-017-0497-9) contains supplementary material, which is available to authorized users.

## Background

Allogeneic umbilical cord blood transplantation (CBT) is a treatment option for many patients with acute myeloid (AML) or acute lymphoblastic (ALL) leukemia who lack an HLA-matched donor [1–4]. In the last two decades, the development of reduced-intensity conditioning (RIC) regimens for CBT has allowed extending its use to patients who were deemed ineligible for myeloablative (MAC) conditioning because of older age or medical comorbidities [5–11]. We recently compared outcomes of AML or ALL patients given CBT after RIC (*n* = 415) versus MAC (*n* = 479) regimens. We observed that, in comparison to MAC patients, RIC recipients had a higher incidence of disease relapse and a lower nonrelapse mortality (NRM), translating to comparable leukemia-free (LFS), GVHD-free, relapse-free survival (GRFS), and overall (OS) survival [11].

Previous studies have demonstrated poor outcomes in patients receiving CB graft containing <2.5 × 10^7^ total nucleated cells (TNC) per kilogram at cryopreservation, particularly in the presence of human leukocyte antigen (HLA)-mismatches [12]. Unfortunately, many adult patients lack a sufficiently rich CB unit to allow safe CBT. Based on these observations, the Minnesota group pioneered the infusion of two CB units from different partially HLA-matched donors (dCBT) for patients who lack a sufficiently rich single CB unit [13]. Based on preliminary encouraging results, this approach has been extended to patients who had a single CB unit containing >2.5 × 10^7^ total nucleated cells (TNC) per kilogram at cryopreservation [14]. This has been particularly the case in the setting of RIC-CBT since it was hypothesized that in comparison with single CBT, double CBT might promote engraftment and increase graft-versus-leukemia effects [15]. The later might be due at least in part via graft-versus-graft alloreactivity as recently demonstrated [16].

In a previous study, we compared transplantation outcomes of adult AML or ALL patients transplanted with one single CB or two CB units after myeloablative conditioning regimen (*n* = 239) [17]. Among patients transplanted with one single CB unit (sCBT), those receiving a thiotepa, busulfan, and fludarabine (TBF) regimen had better LFS than those transplanted with busulfan- or total body irradiation (TBI)-based regimens. When the sCBT group was restricted to patients given TBF-based conditioning, transplantation outcomes were comparable between patients receiving sCBT or dCBT, with the exception for a higher incidence of grade II–IV acute GVHD in dCBT recipients. Similarly, two recent prospective randomized studies demonstrated that dCBT following myeloablative conditioning failed to improve transplantation outcomes in comparison to sCBT in children and/or young adult patients who had a sufficiently rich single CB unit [18, 19].

In the current registry study, we investigated whether these observations remained true in the setting of adults after RIC CBT, which depends primarily on engraftment of donor immune cells and on graft-versus-leukemia effects for disease eradication.

## Methods

### Data collection

This survey is a retrospective, multicenter registry-based study performed by the Acute Leukemia Working Party (ALWP) of the European Society for Blood and Marrow Transplantation (EBMT) and by Eurocord. EBMT registry is a voluntary working group of more than 500 transplant centers, participants of which are required once a year to report all consecutive stem cell transplantations and follow-up. Audits are routinely performed to determine the accuracy of the data. Eurocord collects data on CBT performed in >50 countries worldwide and >500 transplant centers, mainly EBMT centers. Inclusion criteria were adult (≥18 years) patients, AML or ALL, complete remission (CR) at the time of transplantation, first single (with a cryopreserved TNC ≥2.5 × 10^7^/kg) or double CBT between 2004 and 2014, and RIC conditioning. RIC was defined as use of fludarabine associated with <6 Gy TBI, or busulfan ≤8 mg/kg, melphalan ≤140 mg/m^2^ or other nonmyeloablative drugs, as previously reported [11, 20, 21]. HLA-compatibility requirements followed the current practice of antigen level typing for HLA-A and -B and allele level typing of HLA-DRB1. CB units were 4–6/6 HLA-A, -B, and -DRB1 matched to the recipient and to the other unit in case of dCBT in most patients. However, more recently, some centers are no longer matching the CB units between them with regard to HLA based on the study by Avery et al. [22]. HLA disparities between each unit and the recipient and between the two units were not necessarily at the same loci. Grading of acute and chronic GVHD was performed using established criteria [23].

For the purpose of this study, all necessary data were collected according to EBMT and Eurocord guidelines.

### Statistical analyses

Data from all patients meeting the inclusion/exclusion criteria were included in the analyses. Start time was date of transplant for all endpoints. Neutrophil engraftment was defined as first of three consecutive days with a neutrophil count of at least 0.5 × 10^9^/L. Platelet engraftment was defined as the first of seven consecutive days of an unsupported platelet count of at least 20 × 10^9^/L [2].

To evaluate the relapse incidence, patients dying either from direct toxicity of the procedure or from any other cause not related to leukemia were censored. NRM was defined as death without experiencing disease recurrence. Patients were censored at the time of relapse or of the last follow-up. Cumulative incidence functions were used for relapse incidence and NRM in a competing risk setting since death and relapse were competing together.

For estimating the cumulative incidence of chronic GVHD, death was considered as a competing event. OS and LFS were estimated using the Kaplan-Meier estimates. GRFS was defined as being alive with neither grade III–IV acute GVHD, severe chronic GVHD nor disease relapse [24]. Univariate analyses were done using Gray’s test for cumulative incidence function and log rank test for OS and LFS. Associations between single or double CBT and transplantation outcomes (chronic GVHD, relapse, NRM, LFS, and OS) were evaluated in multivariable analyses, using Cox proportional hazards. Variables introduced in the Cox models included recipient age (in decades), disease type (AML versus ALL), disease status at CBT, type of conditioning regimen (TBI, fludarabine, and cyclophosphamide (TCF) versus other), cytogenetic risk, and the use of ATG or not. Exploratory analyses of the heterogeneity of sCBT versus dCBT among pre-transplant subgroups for relapse, NRM, OS, LFS, and GRFS were performed using Cox models. The results of these Cox models were presented graphically using forest plots [25].

All tests were two sided. The type I error rate was fixed at 0.05 for determination of factors associated with time to event outcomes. Statistical analyses were performed with SPSS 19 (SPSS Inc., Chicago, IL), and R 2.13.2 (R Development Core Team, Vienna, Austria) software packages.

## Results

### Patient, disease, and transplant characteristics

Patients and disease characteristics are described in Table 1. Briefly, data from 534 patients with AML (*n* = 408) or ALL (*n* = 126) receiving a first single (*n* = 172) or double (*n* = 362) CBT were included in the analyses. Among dCBT recipients, 47 received two CB units containing less than 2.5 × 10^7^ TNC/kg each. In comparison to sCBT patients, dCBT recipients had a shorter follow-up (34 versus 54 months, *P* = 0.0005), were more frequently male (56 versus 41%, *P* = 0.002), received a conditioning combining TBI, cyclophosphamide and Flu (TCF regimen, 83 versus 66%, *P* < 0.001) more frequently, and received ATG less frequently (16 versus 37%, *P* < 0.001). The two groups were not different for recipient age at transplantation (52 versus 50 years, *P* = 0.17) as well as for other important factors such as disease type (76% of the patients with AML in both group), disease status at transplantation (CR1 in 57 versus 53% of the patients, *P* = 0.6), time from diagnosis to transplantation (9.4 versus 9.5 months, *P* = 0.8) and cytogenetic risks (*P* = 0.85). Finally, as expected, TNC (median 5.1 versus 3.8 × 10^7^ TNC/kg, *P* < 0.001) and CD34^+^ cell (median 4.0 versus 3.1 × 10^5^ cell/kg, *P* = 0.003) doses at cryopreservation were significantly higher in dCBT than in sCBT recipients (Table 2).

**Table 1:** Patient and transplant characteristics

	sCBT (*n* = 172)	dCBT (*n* = 362)	*P* value^a^
Median patient age, months (range)	50 (18–68)	52 (18–76)	0.17
Median follow-up, months (range)	54 (1–118)	34 (2–98)	<0.001
Year of transplantation, median (range)	2008 (2004–2014)	2010 (2005–2014)	<0.001
Recipient sex M, no. (%)	71 (41)	201 (56)	0.002
Recipient weight, median (range)	64	70	<0.001
Time from diagnosis to CBT (months), median (range)			
CR1	6 (3–70)	6 (2–147)	0. 7
CR2	22 (4–95)	22 (6–209)	0.7
Disease, no. (%)			
Acute myeloid leukemia	131 (76)	277 (76)	0.9
Acute lymphoblastic leukemia	41 (24)	85 (24)	
Donor CMV seropositive, no. (%)	107 (65)	222 (64)	0.92
Status at transplantation, no. (%)			
CR1	91 (53)	207 (57)	0.6
CR2	72 (42)	139 (38)	
CR3	9 (5)	16 (4)	
Cytogenetics, no. (%)			0.85
Acute myeloid leukemia			
Good risk^b^	7 (5)	21 (8)	
Intermediate risk^c^	82 (62)	172 (62)	
High risk^d^	20 (15)	33 (12)	
Not reported/failed	22 (17)	51 (18)	
Acute lymphoblastic leukemia			
Intermediate risk^e^	15 (37)	31 (36)	
High risk^f^	18 (44)	38 (45)	
Not reported/failed	8 (19)	16 (19)	
Conditioning regimen, no. (%)			<0.001
TCF	113 (66)	300 (83)	
TBF	19 (11)	5 (1)	
TTBF	9 (5)	0	
FM+/−C	3 (2)	12 (3)	
CF+/−T	9 (5)	5 (1)	
Other	19 (11)	38 (10)	
Missing	0	2 (0.5)	
Recipient CMV-seronegative, no. (%)	57 (35)	123 (36)	0.9
ATG, no. (%)	61 (37)	51 (16)	<0.001
Postgrafting immunosuppression, no. (%)			
CNI + MMF	121 (70)	327 (90)	<0.001
CNI + Pred	21 (12)	4 (1)	
CNI + Mtx	10 (6)	9 (2)	
CNI alone	10 (6)	11 (3)	
Other	10 (6)	11 (3)	

*M* male; *CR* complete remission; *no.* number of patients; *ATG* anti-thymocyte globulins; *TNC* total nucleated cells; *TCF* total body irradiation (TBI), cyclophosphamide and fludarabine; *TBF* Thiotepa, busulfan, and fludarabine; *TTBF* TBI, Thiotepa, busulfan, and fludarabine; *FM+/-C* fludarbine, melphalan with or without cyclophosphamide; *CF+/-T* cyclophosphamide, fludarabine with or without thiothepa; *CNI* calcineurin inhibitor (cyclosporine A or tacrolimus); *MMF* mycophenolate mofetil; *MTX* methotrexate; *Pred* predisolone

^a^Calculated with χ^2^ statistics for categorical variables and Mann-Whitney test for continuous variables

^b^Defined as *t*(8;21), *t*(15;17), inv or del (16), or acute promyelocyticleukemia, these abnormalities only or combined with others

^c^Defined as all cytogenetics not belonging to the good or high risk (including trisomias)

^d^Defined as 11q23 abnormalities, complex caryotype, and abnormalities of chromosomes 5 and 7

^e^Defined as *t*(9;22), *t*(4;11), *t*(8;14), *t*(14;18), low hypodiploidy (30–39 chromosomes)/near triploidy (60–78 chromosomes), and complex karyotype

^f^All others

**Table 2:** Graft characteristics

	sCBT (*n* = 172)	dCBT (*n* = 362)	*P* value^1^
Number of HLA disparities, no. (%)			
0–1 Mismatch	55 (32)	79 (22)	0.13
2 Mismatches	99 (58)	192 (53)	
3–4 Mismatches	9 (5)	24 (7)	
Missing data	9 (5)	67 (18)	
ABO group, no. (%)			0.08
Compatible or minor mismatch	89 (52)	159 (44)	
Major mismatch	53 (31)	139 (38)	
Missing data	30 (17)	64 (18)	
TNC at collection × 10^7^/kg			
Median (range)	3.8 (2.5–9.0)	5.1 (1.5–13.7)^a^	<0.001
CD34^+^ cell at collection × 10^5^/kg			
Median (range)	3.1 (0.6–6.8)	4.0 (0.4–10.4)	0.003
TNC at infusion × 10^7^/kg			
Median (range)	3.1 (0.6–6.8)	4 (0.4–10.4)	<0.001
CD34^+^ cell at infusion × 10^5^/kg			
Median (range)	1.2 (0.2–4.9)	1.2 (0.1–8.5)	0.5

^a^2 Patients had <2.5 × 10^7^ TNC/kg

### Engraftment and GVHD

Overall, the cumulative incidence of neutrophil engraftment at day 60 was not different in sCBT (median 77%; 95% CI 70–83%) and dCBT (median 83%; 95% CI 78–86%) recipients (*P* = 0.4). The median time to neutrophil engraftment was 19 and 24 days, for sCBT and dCBT, respectively, (*P* < 0.001) (Additional file 1: Figure S1). Similarly, the cumulative incidence of platelet engraftment at 6 months was not different in sCBT (median 65%; 95% CI 57–73%) and dCBT (median 71%; 95% CI 65–76%) recipients (*P* = 0.9).

There was a trend for a lower incidence of grade II–IV acute GVHD (28 versus 36%, *P* = 0.08) in sCBT recipients, but this was no longer the case after adjusting for confounding factors (HR = 1.1, *P* = 0.22). In contrast, incidences of grade III–IV acute (11 versus 13%, *P* = 0.6), chronic (28 versus 36% at 2 years, *P* = 0.2) and extensive chronic (10.6 versus 12% at 2 years, *P* = 0.69) GVHD were comparable in sCBT and dCBT recipients.

### Relapse, NRM, GRFS LFS, and OS

At 2 year, in comparison to sCBT recipients, dCBT had a similar cumulative incidence of relapse (32 versus 35%, *P* = 0.5) and of NRM (22 versus 29%, *P* = 0.2). GRFS (37 versus 31%, *P* = 0.13), and LFS (46 versus 36%, *P* = 0.06) were similar according to the type of graft. DCBT showed a significantly better OS (51 versus 41%, *P* = 0.03) (Additional file 1: Table S1 and Figure S2). Of note, outcomes of the 47 patients given 2 CB units containing <2.5 × 10^7^ TNC/kg each were at least as good as those observed in sCBT recipients with 2-year OS and LFS of 56.4 and 42.9%, respectively, (Additional file 1: Figure S3).

**Fig. 1:**
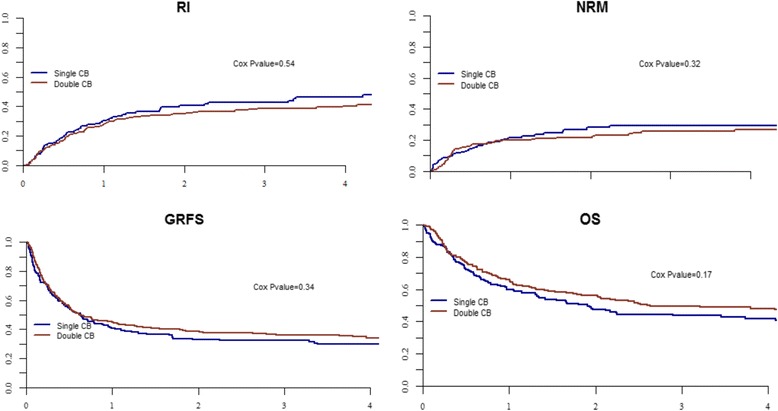
CBT outcomes in acute leukemia patients transplanted following RIC with one (sCBT, *n* = 172) or a two (dCBT, 362) CB unit(s). The figures show the unadjusted curves for sCBT patients and the adjusted curves for dCBT recipients. Curves were adjusted for age at transplantation (in decades), CR2 versus CR1, AML versus ALL, TCF conditioning versus other, ATG, cytogenetic poor versus good/intermediate, and cytogenetic missing versus good/intermediate. *GRFS* GVHD-free relapse-free survival, *OS* overall survival, *RI* relapse incidence, and *NRM* nonrelapse mortality

After adjusting for potential confounding factors in multivariate analyses, dCBT and sCBT recipients had a similar risk of relapse (HR = 0.9; 95% CI 0.6–1.3, *P* = 0.5) and NRM (HR = 0.8; 95% CI 0.5–1.2, *P* = 0.3), and similar GRFS (HR = 1.0; 95% CI 0.9–1.0, *P* = 0.3), LFS (HR = 0.8; 95% CI 0.7–1.1, *P* = 0.2), and OS (HR = 0.8; 95% CI, 0.6–1.1 *P* = 0.17) (Fig. 1) (Table 3). The only factor associated with lower OS in multivariate analysis was the use of ATG (HR = 1.8; 95% CI 1.2–2.8, *P* = 0.01). This was due to a significantly higher NRM in ATG in comparison with non-ATG recipients (HR = 2.4; 95% CI 1.3–4.3, *P* < 0.001) while relapse incidence was not affected by ATG (HR = 1.0, 95% CI 0.6–1.9, *P* = 1.0).

**Table 3:** Outcomes in dCBT versus sCBT in multivariate analyses (adjusted for patient age, disease status, ALL versus AML, conditioning regimen, cytogenetic risk, and ATG)

	Hazard ratio	95% confidence interval		*P* value
		Lower limit	Upper limit	
Relapse	0.90	0.63	1.27	0.54
Nonrelapse mortality	0.81	0.54	1.22	0.32
Leukemia-free survival	0.84	0.65	1.10	0.20
GVHD-free relapse-free survival	1.0	0.9	1.0	0.34
Overall survival	0.83	0.63	1.09	0.17

As shown in the Table 4 and in the Additional file 1: Table S2, causes of death were not statistically different between sCBT and dCBT recipients. However, there was a suggestion for more deaths from GVHD (13/362 (3.5%) versus 3/172 (1.7%)) in dCBT than in sCBT recipients in the first 100 days after transplantation, while the incidence of death from infection in the first 100 days after CBT was comparable between the 2 groups (22/362 (6.1%) in dCBT versus 13/172 (7.6%) in sCBT recipients, respectively).

**Table 4:** Causes of death the first 100 days after CBT (*P* = 0.41)

	sCBT (*n* = 29)	dCBT (*n* = 59)
Relapse or disease progression	6 (20.7)	13 (22.4)
GvHD	3 (10.3)	13 (22.4)
Idiopathic pneumonia syndrome	1 (3.4)	2 (3.4)
Hemorrhage	1 (3.4)	1 (1.7)
Rejection	0 (0.0)	1 (1.7)
Bacterial infection	3 (10.3)	8 (13.8)
Viral infection	0 (0.0)	3 (5.2)
Fungal infection	1 (3.4)	4 (6.9)
Unknown infection	9 (31.0)	7 (12.1)
Cardiac toxicity	0 (0.0)	1 (1.7)
ARDS	1 (3.4)	0 (0.0)
Secondary malignancy	1 (3.4)	0 (0.0)
Multiorgan failure	0 (0.0)	1 (1.7)
LPTD EBV	0 (0.0)	0 (0.0)
Other	3 (10.3)	4 (6.9)
Missing	0	1

### Subgroup analyses

**Fig. 2:**
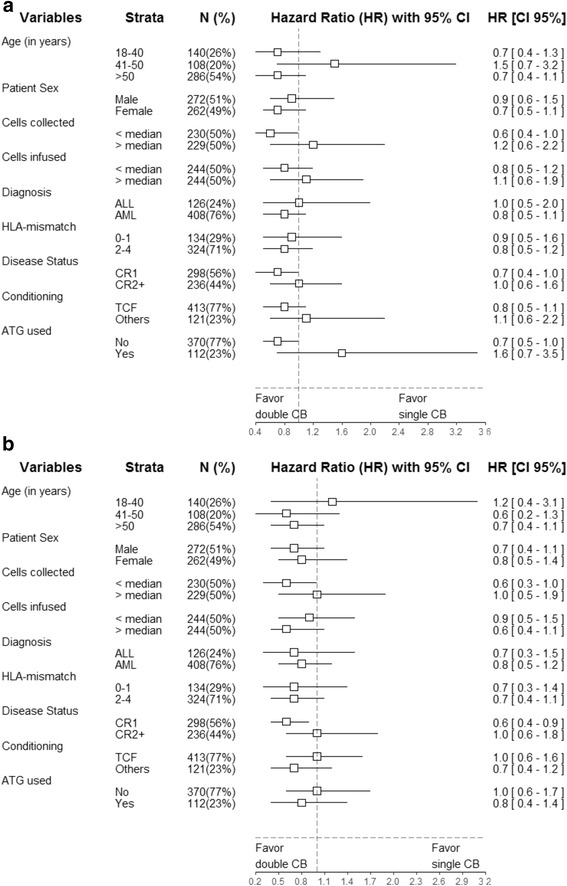
Forest plot analysis of cumulative relapse **a** and nonrelapse mortality **b**. HR and 95% confidence intervals were computed using univariate Cox analyses

**Fig. 3:**
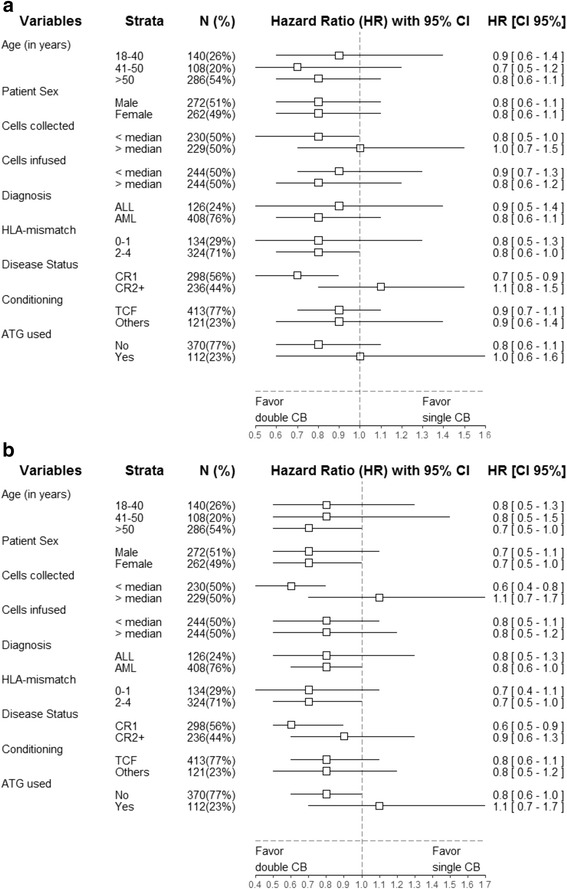
Forest plot analysis of GVHD-free relapse-free survival **a** and overall survival **b**. HR and 95% confidence intervals were computed using univariate Cox analyses

To further dissect the impact of sCBT versus dCBT, we performed additional (univariate) Cox analyses separately for various pre-transplant/transplant variables. The results of these analyses are presented graphically using Forest plots in Figs. 2 and 3. There were no interactions between patient age at transplantation, patient gender, number of cells infused, disease type, disease status, HLA-matching and conditioning type (TCF versus other), and the association between sCBT versus dCBT and GRFS or OS. Further multivariate Cox models assessing possible interactions between ATG and sCBT versus dCBT demonstrated the absence of statistically significant interactions for relapse incidence (*P* = 0.27), NRM (*P* = 0.37), LFS (*P* = 0.97), and OS (*P* = 0.59). Similarly, there was no interaction between disease status (CR1 versus other) and the impact of sCBT versus dCBT on GRFS (*P* = 0.61).

### Impact of cell dose

We finally assessed what was the combined impact of cell dose and sCBT versus dCBT. In order to address this issue, we performed multivariate Cox models including four graft type groups: sCBT and TNC above median, dCBT and TNC above median, sCBT and TNC below median, and dCBT and TNC below median. As observed in the Table 5, in comparison to the reference group (sCBT and TNC above median), patients given sCBT with TNC below the median had a higher risk of relapse (HR = 2.0, 95% CI 1.0–3.9, *P* = 0.04) and a suggestion for a worse LFS (HR = 1.5, 95% CI 0.9–2.3, *P* = 0.11), while outcomes were comparable between patients receiving sCBT and TNC above median and those given dCBT (irrespective of the cell dose received).

**Table 5:** Outcomes in dCBT versus sCBT according to cell dose in multivariate analyses (adjusted for patient age, disease status, ALL versus AML, conditioning regimen, cytogenetic risk, and ATG)

	Hazard ratio	95% confidence interval		*P* value
		Lower limit	Upper limit	
Relapse				
sCBT and TNC > median (ref)	–	–	–	–
dCBT and TNC > median	1.32	0.67	2.58	0.42
sCBT and TNC < median	1.99	1.02	3.89	0.04
dCBT and TNC < median	1.74	0.88	3.43	0.11
Nonrelapse mortality				
sCBT and TNC > median (ref)	–	–	–	–
dCBT and TNC > median	0.69	0.35	1.36	0.29
sCBT and TNC < median	1.01	0.53	1.93	0.97
dCBT and TNC < median	0.93	0.47	1.83	0.84
Leukemia-free survival				
sCBT and TNC > median (ref)	–	–	–	–
dCBT and TNC > median	0.97	0.61	1.55	0.89
sCBT and TNC < median	1.46	0.92	2.30	0.11
dCBT and TNC < median	1.27	0.79	2.03	0.33
Overall survival				
sCBT and TNC > median (ref)	–	–	–	–
dCBT and TNC > median	0.97	0.60	1.58	0.91
sCBT and TNC < median	1.39	0.86	2.23	0.18
dCBT and TNC < median	1.07	0.65	1.77	0.78

## Discussion

Umbilical CB units contain a limited number of hematopoietic cells. This is unfortunate given that cell dose is one of the main predictive factors for CBT outcomes [26–28]. Transplantation of two CB units has been introduced by investigators from the university of Minnesota to increase the cell dose infused [13, 29]. Preliminary studies have demonstrated that this strategy allowed safe CBT in adult patients who lacked a sufficiently rich CB unit [30]. Further studies observed that dCBT induced graft-versus-graft reactions that could increase alloreactivity and perhaps graft-versus-leukemia effects [15]. This prompted us to compare post-transplantation outcomes in patients with acute leukemia receiving sCBT or dCBT after RIC, a transplantation approach that depends mainly on graft-versus-leukemia effects for tumor eradication [31, 32]. Several observations were made.

A first observation was that indeed, dCBT allowed safe CBT in adult patients who lacked a CB unit containing at least 2.5 × 10^7^ TNC/kg since OS and LFS were at least as good in these patients than in those transplanted with a single CB unit containing ≥2.5 × 10^7^ TNC/kg. This is in concordance with the observations reported by the University of Minnesota [30].

A second observation was that patients who received dCBT had a similar incidence of relapse than those given sCBT. This was also true when comparing the relapse incidence in patients receiving sCBT with TNC > median to those receiving dCBT with TNC > median. These observations suggest that graft-versus-leukemia effects are comparable after sCBT or dCBT. A comparable incidence of relapse in patients receiving sCBT or dCBT has also been observed in recent registry [17, 30, 33] or prospective randomized [18, 19] studies including patients given CBT after myeloablative conditioning. Other approaches to decrease relapse incidence after CBT might include post-transplant administration of disease-targeted medications [34–36] or of chimeric antigen receptor T cells [37].

In multivariate analyses, sCBT and dCBT patients had comparable NRM, LFS, GRFS, and OS. These observations are also in accordance with those made in patients receiving CBT after myeloablative conditioning [17–19, 30, 33, 38]. Subgroup analyses revealed no interaction between patient age at transplantation, patient gender, number of cells infused, disease type, disease status, HLA-matching, use of ATG and conditioning type (TCF versus other), and the associations between sCBT versus dCBT and GRFS or OS.

The current study also confirmed a detrimental impact of ATG on NRM (leading to a significantly inferior OS) as recently reported in a study including data from patents given CBT after myeloablative conditioning [39] or RIC dCBT [40]. Further, despite ATG not only induces in vivo T cell depletion of the graft but also promotes the generation of regulatory T cells [41, 42], ATG failed to prevent chronic GVHD in the current study, in contrast to what has been observed in peripheral blood stem cell recipients [43–45]. These results are also in accordance with those reported by Admiraal et al. who demonstrated that reducing the exposure of ATG after CBT (allowing early CD4+ T cell recovery) improved outcomes in pediatric CBT [46].

There are some limitations in our study including its design (retrospective registry survey) and the relative imbalance in the two groups such as more frequent use of the TCF conditioning regimen but less frequent use of ATG in dCBT patients. These differences were carefully adjusted for in multivariate analyses. Another potential limitation of the study is a potential lack of statistical power to detect small advantages of one group to another. However, the number of patients included in the current study (*n* = 534) is higher than the number of patients included in prior registry studies in adults (*n* = 409 in the CIBMTR study [30] and *n* = 239 in the Eurocord/EBMT study [17]) or in recent prospective randomized studies in children (*n* = 224 in the study reported by Wagner et al. [18] and *n* = 151 in the study reported by Michel et al. [19]). Nevertheless, further prospective randomized studies in the RIC setting are needed to draw definitive conclusions. Finally, further studies should compare outcomes after CBT or HLA-haploidentical transplantation following RIC regimens [47–49].

## Conclusions

In summary, we observed comparable outcomes in patients given dCBT or sufficiently rich sCBT with a TNC dose at cryopreservation >2.5 × 10e7/Kg. Recent advances in the field of CBT expansion are likely to improve outcomes of RIC sCBT [50].

## Additional file

Additional file 1: Supplemental figures 1–3 and Tables 1–2. (DOCX 670 kb)

## Abbreviations

ALL: Acute lymphoblastic leukemia
AML: Acute myeloid leukemia
ATG: Anti-thymocyte globulin
CB: Umbilical cord blood
CBT: Umbilical cord blood transplantation
CI: Confidence interval
CR: Complete remission
dCBT: Double-unit cord blood transplantation
EBMT: European Society for Blood and Marrow Transplantation
GRFS: GVHD-free, relapse-free survival
GVHD: Graft-versus-host disease
HLA: Human leukocyte antigen
HR: Hazard ratio
LFS: Leukemia-free survival
NRM: Nonrelapse mortality
OS: Overall survival
RIC: Reduced-intensity conditioning
sCBT: Single-unit cord blood transplantation
TBF: Conditioning regimen combining thiotepa, busulfan and fludarabine
TBI: Total body irradiation
TCF: Conditioning regimen combining TBI, cyclophosphamide and fludarabine
TNC: Total nucleated cells
