# Supplementary material for: Single- or double-unit UCBT following RIC in adults with AL: a report from Eurocord, the ALWP and the CTIWP of the EBMT
Source: J Hematol Oncol. 2017 Jun 21;10:128. doi: 10.1186/s13045-017-0497-9 (PMC5479038; doi:10.1186/s13045-017-0497-9)
Supplement: Additional file 1: — Supplemental figures 1–3 and Tables 1–2. (DOCX 670 kb) [file 13045_2017_497_MOESM1_ESM.docx]

**Supplemental Figure 1. Time to achieve 0.5 x 10^9^ neutrophils/L in acute leukemia patients transplanted following RIC with one (sCBT, n=172) or a two (dCBT, 362) CB unit(s)**.

**Supplemental Figure 2.** CBT outcomes in acute leukemia patients transplanted following RIC with one (sCBT, n=172) or a two (dCBT, 362) CB unit(s).

**Supplemental figure 3.** OS and LFS in sCBT recipients, dCBT recipients receiving at least one CB unit containing > 2.5 x 10^7^ TNC/kg, and dCBT recipients receiving two CB units containing < 2.5 x 10^7^ TNC/kg each.

**Supplemental table 1.** Unadjusted transplantation outcomes

|  | **sCBT**  **(n=172)** | **dCBT**  **(n=362)** | **P value^1^** |
| --- | --- | --- | --- |
| **Relapse incidence** |  |  | 0.5 |
| Incidence at 2-year | 35 | 32 |  |
| Median time (days) (95% CI) | 182 [21-1540] | 181 [10-1770] |  |
| **Nonrelapse mortality** |  |  | 0.2 |
| Incidence at 2-year | 29 | 22 |  |
| Median time (days) (95% CI) | 108 [1-817] | 83 [2-1388] |  |
| **Leukemia-free survival** |  |  | 0.06 |
| Incidence at 2-year | 36 | 46 |  |
| Median time (years) (95% CI) | 1.0 [0.7-1.7] | 2.2 [1.2-4.9] |  |
| **GVHD and relapse-free survival** |  |  |  |
| Incidence at 2-year | 31 | 37 |  |
| Median time (years) (95% CI) | 0.6 [0.4-1.0] | 0.6 [0.5-1.1] |  |
| **Overall survival** |  |  | 0.03 |
| Incidence at 2-year | 41 | 51 |  |
| Median time (years) (95% CI) | 1.6 [1.0-2.9] | 2.7 [2.0-NA] |  |

**Supplemental table 2. Causes of death 100 days – 3 years after CBT (P=0.74).**

|  | **sCBT (n=66)** | **dCBT (n=96)** |
| --- | --- | --- |
|  |  |  |
| Relapse or disease progression | 35 (54.7) | 60 (63.2) |
| GvHD | 6 (9.4) | 10 (10.5) |
| Idiopathic pneumonia syndrome | 1 (1.6) | 0 (0.0) |
| Hemorrhage | 1 (1.6) | 1 (1.1) |
| Rejection | 0 (0.0) | 1 (1.1) |
| Bacterial infection | 1 (1.6) | 3 (3.2) |
| Viral infection | 5 (7.8) | 6 (6.3) |
| Fungal infection | 2 (3.1) | 2 (2.1) |
| Unknown infection | 6 (9.4) | 4 (4.2) |
| Cardiac toxicity | 1 (1.6) | 0 (0.0) |
| ARDS | 0 (0.0) | 1 (1.1) |
| Secondary malignancy | 0 (0.0) | 0 (0.0) |
| Multiorgan failure | 1 (1.6) | 2 (2.1) |
| LPTD EBV | 1 (1.6) | 3 (3.2) |
| Other | 4 (6.2) | 2 (2.1) |
| Missing | 2 | 1 |

**List of participating centers**

Hopital Saint Antoine, Department of Hematology, Paris, France; Programme de Transplantation&Therapie Cellulaire, Centre de Recherche en Cancérologie de Marseille, Institut Paoli Calmettes, Marseille, France; Erasmus MC Cancer Institute, University Medical Center Rotterdam, Department of Hematology, Rotterdam, The Netherlands; CHU Nantes, Dept. D`Hematologie, Nantes, France; University Hospital La Fe, Hematology Department (Torre F, Planta 7), Valencia, Spain; University Medical Centre, Dept. of Haematology, Utrecht, The Netherlands; CHU Lapeyronie, Département d`Hématologie Clinique, Montpellier, France; CHU Bordeaux, Hôpital Haut-leveque, Pessac, France; Hopital La Miletrie, Head of the Bone Marrow TransplantUnit, Clinical Hematology, Poitiers, France; Hôpital Percy, Hematology Department, Clamart, France; Centre Hospitalier Lyon Sud, Service Hematologie, Lyon, France; Gustave Roussy, institut de cancérologie, BMT Service, Division of Hematology,, Department of Medical Oncology, Villejuif, France; Hopital Jean Minjoz, Service d'Hématologie, Besancon, France; Medical University Graz, LKH - University Hospital Graz, Division of Haematology, Graz, Austria; CHU Nice - Hôpital de l'ARCHET I, Hematologie Clinique, Nice, France; Hopital Henri Mondor, Sve d` Hematologie, Creteil, France; Bone Marrow Transplant Unit L 4043, National University Hospital, Rigshospitalet, Copenhagen, Denmark; Hopital St. Louis, Dept.of Hematology - BMT, Paris, France; CHU CAEN, Institut d’hématologie de Basse-Normandie, Caen, France; Leiden University Hospital, BMT Centre Leiden, Leiden, The Netherlands; Azienda Ospedaliera Universitaria Careggi, Cell Therapy and Transfusion Medicine Unit, Firenze, Italy; Hopital d`Enfants, Hematology, Vandoeuvre Les Nancy, France; Royal Marsden Hospital, Leukaemia Myeloma Units, London, United Kingdom; Hopital A. Michallon, Department of Hematology, Grenoble, France; Hôpital HURIEZ, UAM allo-CSH, CHRU, Lille, France; VU University Medical Center, Department of Hematology (Br 250), Amsterdam, The Netherlands; Nottingham University, Hucknall Road, Nottingham, United Kingdom; GKT School of Medicine, Dept. of Haematological Medicine, London, United Kingdom; CHRU St. Etienne, Hopital Nord, Service d`Hematologie Clinique, Saint Etienne, France; CHU ESTAING, Service d’hématologie clinique Adulte et pédiatrie, Clermont-Ferrand, France; Bristol Royal Hospital for Children, Dept. of Paediatric Oncology/BMT, Bristol, United Kingdom; Klinikum Grosshadern, Med. Klinik III, Munich, Germany; Yorkshire Blood & Marrow Transplant Programme, Haematology Department, Level 3, Bexley Wing, St James`s Institute of Oncology,, Leeds, United Kingdom; University Hospital Maastricht, Dept. Internal Med.Hematology /Oncology, Maastricht, The Netherlands; Institut Universitaire du Cancer Toulouse, Oncopole, Toulouse, France; Chaim Sheba Medical Center, Chaim Sheba Medical Center, Dept. of Bone Marrow Transplantation, Tel-Hashomer, Israel; Azienda Ospedali Riuniti di Ancona, Department of Hematology, Ancona University, Ancona, Italy; University Hospital Erlangen, Dept. of Internal Medicine 5, Erlangen, Germany; Centre Henri Becquerel, Hematology, Rouen, France; Bone Marrow Transplant Unit, Beatson, West of Scotland Cancer Centre, Gartnaval General Hospital, Glasgow, United Kingdom; Department of Haematology, Cancer and Haematology Centre, Churchill Hospital, Oxford, United Kingdom; University Hospital Birmingham NHSTrust, Queen Elizabeth Medical Centre, Edgbaston, Dept. of Haematology, Birmingham, United Kingdom; Beilinson Hospital, Hematology and BMT Department, Petach-Tikva, Israel; ICO-Hospital Universitari Germans Trias i Pujol, Cattedra e Servizio di Ematologia, Barcelona, Spain; C.H.R.U de Brest, Service Onco-Hematologie, Brest, France; Centre Hospitalier Universitaire de Rennes, Service d`Hematologie Clinique Adulte, Rennes, France; Universitaetsklinikum Wuerzburg, Med. Klinik und Poliklinik II, Wuerzburg, Germany; Ospedale San Raffaele s.r.l., Haematology and BMT, Milano, Italy; Hôpital Necker, Service Hematologie Adulte, Paris, France; Hospital Sirio-Libanes, Hematology Bone Marrow Transplant Unit, Sao Paulo, Brazil; Nijmegen Medical Centre, Department of Hematology, Nijmegen, The Netherlands; Universite Paris IV, Hopital la Pitié-Salpêtrière, Hematologie Clinique, Paris, France; Hospital Clínico Universitario, Servicio de Hematología, Valencia, Spain; Ospedale S. Camillo-Forlanini, Dept. of Hematology and BMT, Rome, Italy; Universita Cattolica S. Cuore, Istituto di Ematologia, Ematologia, Rome, Italy; Manchester Royal Infirmary, Clinica Haematology Department, Manchester, United Kingdom; Evangelismos Hospital, Division  of Hematology, BMT Unit, Athens, Greece; North Trent BMT Programme (Adults), Sheffield Teaching Hospitals NHS Trust, Royal Hallamshire Hospital, Sheffield, United Kingdom; CHRU Limoges, Service d`Hématologie Clinique, Limoges, France; Klinikum Augsburg, II Medizinische Klinik, Augsburg, Germany; University Hospital Gasthuisberg, Dept. of Hematology, Leuven, Belgium; Karolinska University Hospital, Dept. of Hematology, Stockholm, Sweden; Medizinische Universitaet Wien, Klinik fuer Innere Medizin I, Knochenmarktransplantation, Vienna, Austria; Hospital Santa Creu i Sant Pau, Hematology Department, Barcelona, Spain; Hannover Medical School, Department of Haematology, Hemostasis, Oncology, and Stem Cell Transplantation, Hannover, Germany; Klinikum Rechts der Isar, III Med Klinik der TU, Munich, Germany; Az. Ospedaliera S. Croce e Carle, Division of Hematology, Cuneo, Italy; University Hospital Eppendorf, Bone Marrow Transplantation Centre, Hamburg, Germany; Universitair Ziekenhuis Brussel, Division of Clinical Hematology, Brussels, Belgium; CHRU, Service des Maladies du Sang, Angers, France; Azienda Ospedaliera Papa Giovanni XXIII, Hematology and Bone Marrow Transplant Unit, Bergamo, Italy; Hospital Clínico, Servicio de Hematología, Salamanca, Spain; St. Bartholomew`s and The Royal London NHS Trust, London, United Kingdom; University Regensburg, Dept. of Hematology and Oncology, Regensburg, Germany; Hospital Guglielmo da Saliceto, Oncology and Hematology Department, Piacenza, Italy; University Hospital, Hematology, Basel, Switzerland; Klinik fuer Innere Medzin III, Universitätsklinikum Ulm, Ulm, Germany; Hospital Clinic, Institute of Hematology & Oncology, Dept. of Hematology, Barcelona, Spain; Univ. La Sapienza, Dip. Biotecnologie Cellulari ed Ematologia, Rome, Italy; Bologna University, S.Orsola-Malpighi Hospital, Institute of Hematology & Medical, Oncology L & A Seràgnoli, Bologna, Italy; Hospital U. Marqués de Valdecilla, Servicio de Hematología-Hemoterapia, Santander, Spain; University Hospital, Dept. of Medicine, Uppsala, Sweden; Adult HSCT unit, Northern Centre for Bone Marrow Transplantation, Freeman Hospital, Newcastle-Upon-Tyne, United Kingdom; Birmingham Heartlands Hospital, Department of Haematology, Birmingham, United Kingdom; Spedali Civili - Brescia, Hematology Division, Department of Medical Oncology, Brescia, Italy; Onco-Ematologia Pediatrica, Centro Trapianti Cellule Staminali, Ospedale Infantile Regina Margherita, Torino, Italy; Rambam Medical Center, Dept. of Hematology & BMT, Haifa, Israel; University Hospital &quot;Queen Johanna-Isul&quot;, Sofia, Bulgaria; Istituto Clinico Humanitas, Transplantation Unit, Department of Oncology and Haematology, Milano, Italy; King Faisal Specialist Hospital & Research Centre, Oncology (Section of Adult Haematolgy/BMT), Riyadh, Saudi Arabia; St. Anna Kinderspital, Stem Cell Transplantation Unit, Vienna, Austria; Hospital Univ. Virgen de las Nieves, Servicio de Hematología, Granada, Spain; George Papanicolaou General Hospital, Haematology Department / BMT Unit, Thessaloniki, Greece; Hospital Clinico Universitario, Servicio de Hematología, Santiago De Compostela, Spain; Hospital Regional de Málaga, Servicio de Hematología, Málaga, Spain; Hospital Vall d`Hebron, Unidad de Adultos, Barcelona, Spain; Elisabethinen-Hospital, I. Internal Department, Linz, Austria; Hôpital Robert Debre, Pediatric Hematology and Immunology Department, Paris, France; Jessa Ziekenhuis, Dept. of Hematology, Hasselt, Belgium; Hospital Universitario Central de Asturias, Oviedo, Spain; Techniciens d`Etude Clinique suivi de patients greffes, Nouvel Hopital Civil, Strasbourg, France; Azienda Ospedaliero Universitaria di Udine, Division of Hematology, P.le S. Maria della Misericordia, Udine, Italy; Leicester Royal Infirmary, Department of Haematology, NHS Trust, Leicester, United Kingdom; Hospital Universitari Son Espases, Hematology Service, Palma De Mallorca, Spain; Umea University Hospital, Hematology, Umeå, Sweden; University Hospital, Dept. of Hematology, Linköping, Sweden; ¨Tor Vergata¨ University of Rome, Stem Cell Transplant Unit, Policlinico Universitario Tor Vergata, Rome, Italy; Christie NHS Trust Hospital, Adult Leukaemia and Bone Marrow Transplant Unit, Manchester, United Kingdom; Medical Park Hospitals, Stem Cell Transplant Unit, Antalya, Turkey.
